# Propofol-induced NGF/CREB suppression and microglial M1 polarization: a correspondence

**DOI:** 10.1097/JS9.0000000000002995

**Published:** 2025-07-08

**Authors:** Yulong Hu, Leilei Hu

**Affiliations:** Department of Anesthesiology, The Second Affiliated Hospital of Nanchang University, Nanchang, China

*Dear Editor,*

We read with great interest the recent study by Ye *et al* titled “**Induction of M1 polarization in BV2 cells by propofol intervention promotes perioperative neurocognitive disorders through the NGF/CREB signaling pathway.**” As artificial intelligence increasingly shapes scientific reporting, we adhered to the 2025 TITAN guidelines on transparency in AI use, acknowledging that no AI tools were employed in preparing this correspondence^[1]^. Their comprehensive investigation uncovers a critical mechanism by which propofol disrupts microglial homeostasis and contributes to perioperative neurocognitive disorders, highlighting the downregulation of the NGF/CREB signaling axis as a pivotal event.

This study is particularly notable for demonstrating that propofol not only inhibits NGF and phosphorylated CREB expression in both BV2 microglial cells and hippocampal tissue, but also induces M1 polarization, oxidative stress, and inflammatory cytokine release—all hallmarks of neurocognitive dysfunction. The reversal of these effects by exogenous NGF further reinforces the functional relevance of this pathway.^[2]^

Importantly, the authors employed both in vitro and in vivo models to convincingly correlate molecular alterations with cognitive outcomes. Their use of RNA interference and NGF supplementation strategies adds mechanistic depth and identifies potential therapeutic avenues.

Propofol is widely used, especially in elderly and high-risk patients. The finding that it may exacerbate neuroinflammation via disruption of the NGF/CREB pathway calls for caution. For instance, a randomized trial showed greater verbal memory decline in elderly patients anesthetized with propofol compared to isoflurane.^[3]^ Given the aging global population and the increasing number of surgeries in patients over 65, understanding propofol’s impact on postoperative cognitive outcomes is clinically urgent.

Moreover, exogenous NGF was shown to mitigate propofol-induced M1 polarization and cognitive decline. Although direct CNS delivery of NGF is impractical, pharmacological NGF mimetics or small molecules that enhance p-CREB activity warrant exploration^[4]^. Drug development targeting neurotrophin pathways may open new avenues in anesthetic safety research, particularly in preventing long-term cognitive impairment.

Another promising direction involves biomarker discovery. Perioperative monitoring of serum NGF or p-CREB levels could serve as early indicators of PND risk and help guide anesthetic choices^[5]^. Incorporating these biomarkers into perioperative risk stratification models may support real-time clinical decision-making, especially in neurocognitively vulnerable patients.

Additionally, the study underscores the importance of considering both anesthetic type and duration. Both single high-dose and repeated administration of propofol impair cognition and promote microglial M1 polarization. Previous studies have confirmed that repeated exposure to propofol activates NF-κB and NLRP3, exacerbating neuroinflammation and memory loss^[6]^. Thus, re-evaluating dose thresholds and considering alternatives such as dexmedetomidine may be warranted in scenarios requiring prolonged or repeated sedation.

Finally, it is essential to acknowledge the context-dependent effects of propofol. While some studies highlight its neuroprotective roles through pathways such as PKA/p-CREB signaling, others demonstrate that in neonatal rodents, propofol downregulates p-CREB and BDNF, impairing spatial memory^[7]^. These contrasting effects underscore the importance of age, dosage, and exposure duration in shaping propofol’s neuroinflammatory profile.

In summary, Ye et al. compellingly link propofol-induced NGF/CREB downregulation to microglial activation and cognitive dysfunction. This provides a robust mechanistic platform for both basic and translational studies and highlights the need for clinical trials evaluating NGF-enhancing strategies and alternative anesthetics—especially for elderly or cognitively vulnerable patients. As the surgical population ages and neuroinflammation emerges as a central factor in cognitive disorders, studying anesthetic-related mechanisms becomes not only scientifically compelling but also clinically essential.

## Data Availability

This manuscript is a comment. Don’t need a data availability statement. However, all the data from the current study are publicly available.
